# Role of Poly(ADP-Ribose) Polymerase (PARP) Enzyme in the Systemic Acquired Acclimation Induced by Light Stress in *Phaseolus vulgaris* L. Plants

**DOI:** 10.3390/plants11141870

**Published:** 2022-07-18

**Authors:** Luca Vitale, Ermenegilda Vitale, Anna Rita Bianchi, Anna De Maio, Carmen Arena

**Affiliations:** 1 Institute for Agricultural and Forestry Systems in the Mediterranean (ISAFoM), National Research Council of Italy (CNR), P. le Enrico Fermi 1, Loc. Porto del Granatello, 80055 Portici, Italy; luca.vitale@cnr.it; 2 Department of Biology, University of Naples Federico II, Via Cinthia, 80126 Naples, Italy; ermenegilda.vitale@unina.it (E.V.); annarita.bianchi@unina.it (A.R.B.)

**Keywords:** high light, stress signaling, PARP, photochemistry, systemic acquired acclimation

## Abstract

Plants are able to acclimate to environmental constraints through functional modifications that may also occur in tissues that are not directly exposed to stress. This process is termed “systemic acquired acclimation.” The present study aims to evaluate the involvement of PolyADP-ribose) polymerase (PARP) protein in the acclimation process to high light (HL) stress in *Phaseolus vulgaris* plants. For this purpose, some leaves located at the top of the plant, in the apical position, were directly exposed to HL (“inducing” leaves), while others on the same plant, distal from the top, continued to be exposed to growth light (“receiving” leaves) to verify the hypothesis that an “alert” message may be transferred from injured tissues to distal ones. Biochemical and eco-physiological analyses, namely PARP activity, H_2_O_2_ and water- and fat-soluble antioxidants (i.e., ascorbic acid, tocopherol, glutathione (GSH), phenols, carotenoids, etc.) content, and chlorophyll fluorescence measurements were performed on both “inducing” and “receiving” leaves. Even if no change in PARP expression was found, its activity increased in “receiving” unstressed leaves in response to the light stress duration experimented by “inducing” leaves, while antioxidant capacity declined. When the “receiving” leaves were exposed to HL, the PARP activity returned to the control value, while antioxidant capacity photosynthetic electron transport rate (J_f_) decreased and increased, respectively, compared to Control. Our results seem to show an acclimation pathway triggered in remote tissues not yet subjected to stress, likely involving a reactive oxygen species wave activating the PARP enzyme in a mechanism still to be clarified. In addition, the increased tolerance of plants directly exposed to HL could implicate a boosted synthesis of soluble antioxidants accompanied by a reduction of PARP activity to reduce excessive consumption of NAD(P).

## 1. Introduction

Plants respond to abrupt environmental changes through molecular, physiological, and structural strategies, allowing plant acclimation to abiotic stress. Rapid systemic signaling between different plant parts or organs during stress is a driving factor for the acclimation. The acclimation in tissues spatially far from those directly exposed to abiotic stress is termed “systemic acquired acclimation” (SAA), and was first described in *Arabidopsis thaliana* [1]. To date, this process has been reported in many other species in response to different abiotic stresses [2,3,4] and different molecules have been identified as possible “messengers” for triggering the systemic signals, including reactive oxygen species (ROS), calcium, and hydraulic waves and electric signals [5,6]. Among the systemic signals, ROS have proven to elicit the acclimation mechanism in remote tissues resulting in the activation of transcriptional responses improving plant tolerance to stress [3].

A new and putative role in SAA could be facilitatedby poly(ADPribose)-polymerase (PARP) enzymes. Members of thePARP family catalyze a covalent and reversible post-translational modification of proteins named Poly(ADP)ribosylation (PAR) [7].

To date, 18 isoforms of PARP have been identified, which differ in molecular weight and cellular localization [8]. Compared to the 18 members of the human PARP superfamily [9,10], plants contain a lower number of such proteins, grouped into three categories [11].

In plants, as in animals, nuclear PARPs are considered “sensors of DNA damage”, because their activation represents the first molecular response to genomic materials oxidative damage [12,13]. PAR plays several cellular and molecular functions in both plant and animal cells, such as DNA repair, modulation of different cell death pathways, regulation of transcription and replication, and chromatin compaction [9].

It is ascertained that plant stress tolerance may implicate a down- or up-regulation of PARPs activity, which confers protection against harmful effects of environmental stress, such as high and low temperatures, ionizing radiation, pH, dehydration, high light, and salinity [11,14,15,16]. It has been demonstrated that the decline of PARP activity implicates a reduced consumption of NAD^+^ and ADP in order to preserve the cell homeostasis avoiding apoptosis. Furthermore, a reduction of PARP may also trigger signaling pathways at transcriptional level active against the stress [17,18].

In this study, we suppose for *Phaseolus vulgaris* L. plants a functional involvement of PARP enzyme in SAA mechanism. To test this hypothesis, we studied the modulation of PARP activity during the systemic acquired acclimation in remote tissue of *P. vulgaris* directly and not directly exposed to high light stress. To our knowledge, the present study is the first aimed to verify the possible involvement of PARP in the SAA mechanism.

Specific aims of this study were: (i) evaluate the PARP activation in leaves not directly exposed to high light stress (named “receiving leaves”); (ii) assess the modulation of PARP activity and non-enzymatic antioxidant capacity during the SAA, and (iii) evaluate the photochemical regulation of photosystem II (PSII) in bottom leaves (named “receiving leaves”) after direct exposure of top leaves to light stress (named “inducing leaves”). We assumed that the ROS wave generated in stressed tissue would trigger an increase of PARP activity in remote tissues that, in turn, would enhance the stress tolerance of inner, distal leaves not directly exposed to high light stress.

## 2. Results

### 2.1. PARP Activity and Expression in “Inducing” and “Receiving” Phaseolus vulgaris L. Leaves Subjected to High Light Stress

**Figure 1:**
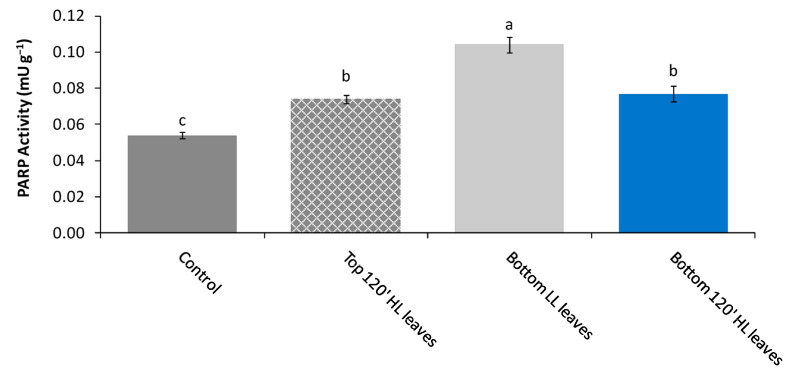
PARP activity in *P. vulgaris* L. Control, Top 120′ HL leaves (inducing top leaves after 120 min of light stress), Bottom LL leaves (receiving bottom unstressed leaves) and Bottom 120′ HL leaves (receiving bottom leaves after 120 min of light stress). Different letters indicate statistically significant differences among treatments (*p* ≤ 0.05) by One-Way ANOVA followed by Holm–Sidak post-hoc test (n = 5).

In the first experiment, the activity (Figure 1) and expression of PARP (Figure S1a) were determined on “inducing” top leaves after 120 min of High Light stress (Top 120′ HL stress), “receiving” not stressed bottom leaves (Bottom 120′ LL leaves), and “receiving” bottom leaves after 120 min of High Light stress (Bottom 120′ HL leaves), then comparing to controls. The western blotting (SDS-PAGE) analysis with anti-PARP antibodies did not show any differences in qualitative and quantitative PARP patterns. Indeed, in all samples, the immunocomplex detection revealed the presence of two bands corresponding to a protein of 62 kDa and 48 kDa, respectively (Figure S1a). However, despite the same protein expression, significant differences in PARP activity were detected among treatments (Figure 1).

The PARP activity of Top and Bottom HL leaves was comparable and significantly higher (about 1.6 times) than Control. The highest PARP activity was detected in Bottom LL leaves (Figure 1). Particularly in the latter, the PARP activity was two-fold higher than Control.

In the second experiment, PARP activity (Figure 1) and expression (Figure S2a) were measured after the application of alight stress of different duration in minutes (‘) (5′, 15′, 30′, 60′, 120′) to “inducing” top leaves (Top HL leaves) and compared to PARP activity and expression of “receiving” unstressed bottom leaves (Bottom LL leaves) and “receiving” stressed bottom leaves (Bottom HL leaves).

**Figure 2:**
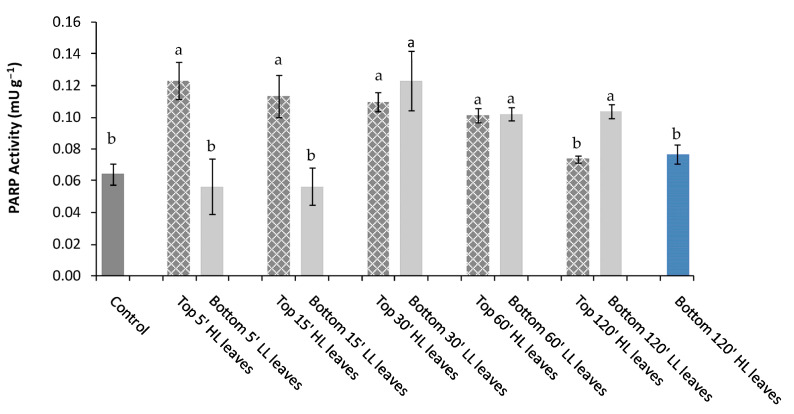
PARP activity in *P. vulgaris* L. Control, Top 5′, 15′, 30′, 60′ and 120′ HL leaves (inducing stressed after 5′, 15′, 30′, 60′ and 120′ of light stress), Bottom LL leaves (receiving unstressed) and Bottom 120′ HL leaves (receiving leaves after 120′ of light stress). Different letters indicate statistically significant difference among treatments (*p* ≤ 0.05) by One-Way ANOVA followed by Holm-Sidak post-hoc test.

As observed in the first experiment, the western blotting analysis showed no significant difference in qualitative and quantitative PARP expression among leaves in response to light stress of different duration, showing all leaves two identical bands of 62 kDa and 48 kDa, respectively (Figure S2a). Conversely, PARP activity was strongly responsive to duration of the light stress (Figure 2). More specifically, after 5′,15′, 30′ and 60′ from the light stress, the PARP activity was higher in Top HL leaves than Control, up to 60′ light stress application on the leaf lamina. On the contrary, it declined after 120′ of stress to values similar to Control (Figure 2). In Bottom LL leaves, no change of PARP activity was observed after 15′ compared to Control, while a significant PARP activity increase was detected after 30′, 60′, and 120′ (Figure 2). Finally, the exposure of Bottom leaves to 120′ of light stress (Bottom HL stress) did not induce significant PARP changes compared to Control.

### 2.2. H_2_O_2_ Content in “Inducing” and “Receiving” Leaves Subjected to High Light Stress

**Figure 3:**
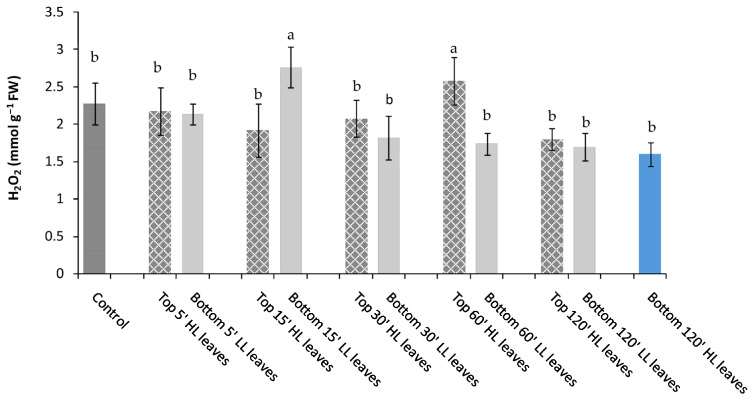
H_2_O_2_ content in *P. vulgaris* L. Control, Top 5′, 15′, 30′, 60′, and 120′ HL leaves (inducing stressed after 5′, 15′, 30′, 60′ and 120′ of light stress), Bottom LL leaves (receiving unstressed) and Bottom 120′ HL leaves (receiving leaves after 120′ of light stress). Different letters indicate statistically significant difference among treatments (*p* ≤ 0.05) by One-Way ANOVA followed by Holm–Sidak post-hoc test.

Besides PARP activity, also endogenous H_2_O_2_ production was determined after the application of a light stress of different duration in minutes (5′, 15′, 30′, 60′, 120′) in “inducing” top leaves (Top HL leaves) and compared to the content measured in “receiving”unstressed bottom leaves (Bottom LL leaves) and “receiving” stressed bottom leaves (Bottom HL leaves) (Figure 3).

Up to 60′ light stress, Top HL leaves showed no significant change in H_2_O_2_ concentration compared to Control, whereas after 120′ of light stress, a significant decline was observed (Figure 3). After 15′ stress, an increase of H_2_O_2_ was measured only in Bottom 15′ LL leaves (Figure 3). After Top leaves were stressed for 30′ (Top 30′ HL leaves), a significant reduction of H_2_O_2_ in Bottom 30′ LL leaves was found, while following Top leaves stress of 60′ and 120′ no further reduction was observed. Moreover, after 120′ light stress on Bottom HL leaves the H_2_O_2_ content was statistically comparable to Top HL and Bottom LL leaves (Figure 3).

### 2.3. Water-Soluble and Fat-Soluble Antioxidant Capacity in “Inducing” Leaves Subjected to High Light Stress and in Unstressed “Receiving” Leaves

**Figure 4:**
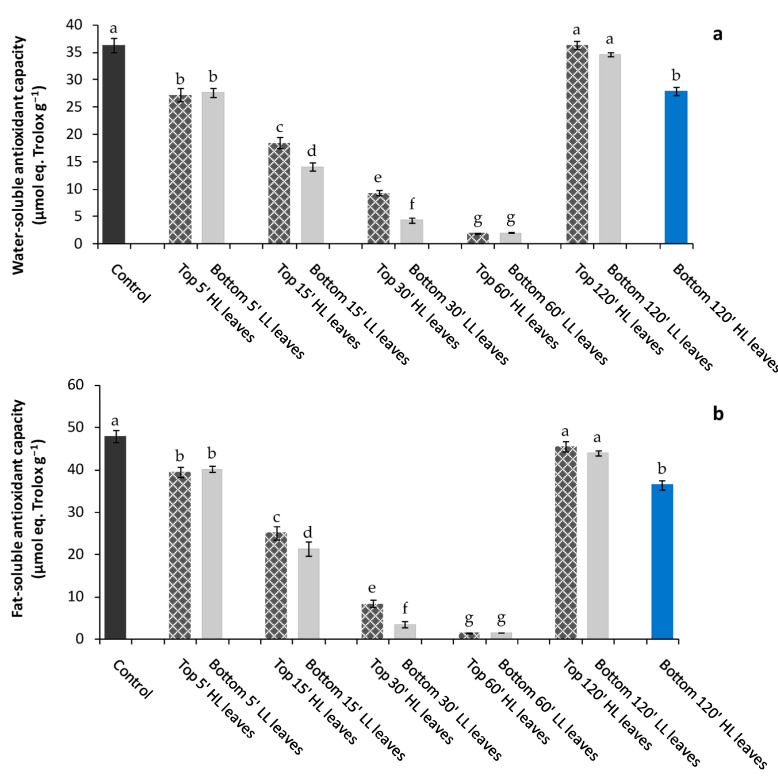
Water-soluble (**a**) and fat-soluble (**b**) antioxidant capacity in *P. vulgaris* L. Control, Top 5′, 15′, 30′, 60′, and 120′ HL leaves (inducing stressed after 5′, 15′, 30′, 60′, and 120′ of light stress), Bottom LL leaves (receiving unstressed) and Bottom 120′ HL leaves (receiving leaves after 120′ of light stress). Different letters indicate statistically significant difference among treatments (*p* ≤ 0.05) by One-Way ANOVA followed by Holm–Sidak post-hoc test.

Total water-soluble (Figure 4a) and fat-soluble (Figure 4b) antioxidant capacity measured in Top HL leaves stressed for 5′, 15′, 30′, 60′, 120′, and in Bottom LL leaves as soon after the different timing of stress was imposed to Top HL leaves strongly declined as the stress duration on Top HL leaves increased.

Interestingly, in unstressed “receiving” leaves, total soluble and fat-soluble antioxidant capacity showed the same behavior of “inducing” stressed leaves, although they did not experiment directly the light stress.

Just after 5′, a significant reduction of soluble and fat-soluble antioxidant capacity in Top HL leaves was found (Figure 4a,b). As stress duration rising, (from 15′ to 30′ to 60′), the water and fat-antioxidant capacity progressively declined in both Top HL and Bottom LL leaves. On the contrary, after 120′ stress imposed to Top “inducing” leaves, total water-soluble and fat-soluble antioxidant pools significantly increased in Top HL and Bottom LL leaves to values measured in Control leaves. Lastly, the light stress of 120 min on Bottom leaves (Bottom 120′ HL leaves), induced a high level of water and fat-soluble antioxidant capacity. However, these values remained lower than Control and Bottom LL leaves when Top HL leaves experimented the stress of the same extent (Figure 4a,b).

### 2.4. Fast Kinetic Fluorescence Light Response Curves in “Inducing” and “Receiving” Leaves Subjected to High Light Stress

**Figure 5:**
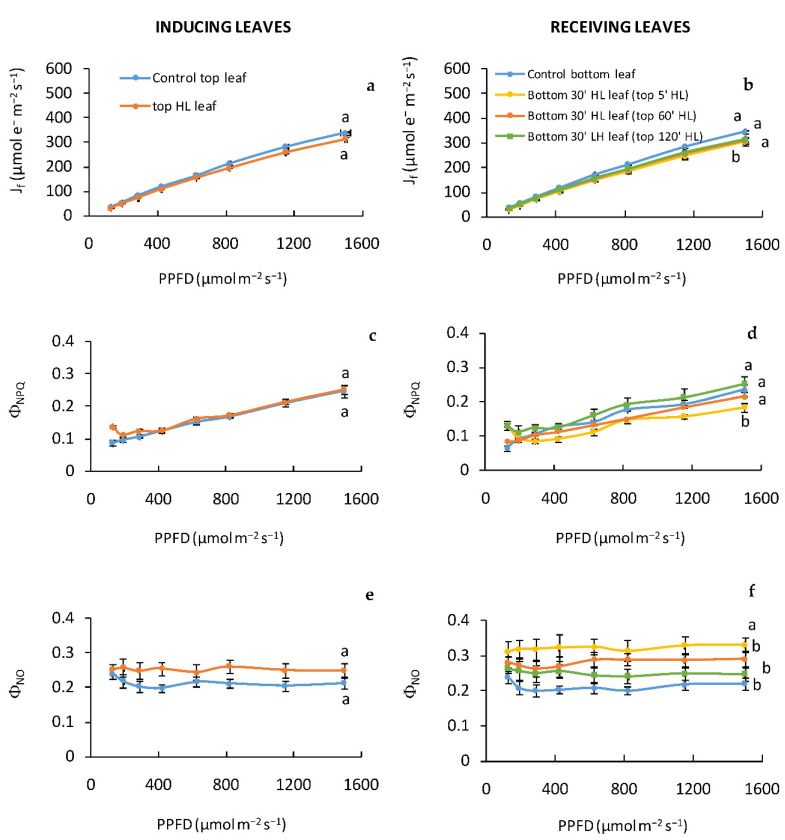
Fast kinetic light response curves: electron transport rate (J_f_) (**a**,**b**), quantum yield of regulated energy dissipation (Φ_NPQ_) (**c**,**d**), quantum yield of non-regulated energy dissipation (Φ_NO_) (**e**,**f**) in *P. vulgaris* L. Control, Top HL “inducing”, and Bottom HL “receiving” leaves. Different letters indicate statistically significant difference among treatments (*p* ≤ 0.05) by One-Way ANOVA followed by Holm–Sidak post-hoc test.

In a third experiment, we exposed the Top “inducing” and Bottom “receiving” leaves to high light for 30′. This exposure determined changes in the regulation of absorbed light energy at PSII (Figure 5). More specifically, similarly to the “inducing” top leaves (Figure 5a,c), the electron transport rate (J_f_) decreased and the regulated-energy dissipation (Φ_NPQ_) increased in the “receiving” bottom leaves (Figure 5b,d). However, the photochemical adjustments were significant in Bottom 30′ HL leaves only after top leaves were exposed to 5′ light stress (Top 5′ HL) (Figure 5b,d,f). More specifically, in these leaves the lowest value of J_f_ and Φ_NPQ_, and the highest value of Φ_NO_ was measured. On the contrary, after top leaves were exposed to a prolonged light stress (Top 60′ and 120′ HL) no significant difference was observed in Bottom 30′ HL stressed leaves for J_f,_,Φ_NPQ_ and Φ_NO_ compared to Control bottom leaves (Figure 5b,d,f).

The maximum PSII photochemical efficiency (F_v_/F_m_) showed the same behavior, being significantly lower in 30′ stressed bottom leaves (Bottom HL leaves) after top leaves were exposed to a light stress of 5′ (Top 5′ HL leaves) compared to Control (Table 1).

## 3. Discussion

Plants are exposed to stress conditions during their growth cycle that may disturb the photosynthetic performance and affect crop yield. Several responses, ranging from morphological to biochemical adjustments, are carried out by plants, and these responses areessential for survivalin their environments. Recent studies focused on the importance of a rapid assessment of stress conditions, allowing plants to rapidly counteract adverse situations in a changing environment. Some plants may use rapid whole-plant systemic signals such as calcium, reactive oxygen species (ROS), and hydraulic and electric waves in mediating plant acclimation during several abiotic stresses. In our experiments, we assessed if, in *P. vulgaris* plants, systemic signaling among leaves is active and can work in counteracting the effects of high light stress on photosynthetic apparatus. To test this hypothesis, we exposed to high light (HL) stress (2000 μmol photons m^−2^ s^−1^) apical top leaves (inducing leaves) and determined, over time, the physiological and biochemical modifications in bottom leaves of the same plant (receiving leaves) that did not directly experience the light stress. After just 5′, a significant reduction of water-soluble andfat-soluble antioxidants occurred in receiving leaves, suggesting that a sort of stress signal, likely ROS wave, was generated from the local spot of HL stress toward remote tissues, namely receiving bottom unstressed leaves [19].

The reduction of water- and fat-soluble antioxidant content observed in bottom receiving leaves (remote tissues) was significant after top leaves experienced 15′ of HL. Under this circumstance a parallel increment of H_2_O_2_ content (considered as a proxy of ROS generation) was found in the receiving leaves, confirming the putative role of H_2_O_2_ as a stress signal triggering a scavenger response by the antioxidant plant system. In response to ROS increase, the soluble antioxidants, scavenging oxidizable substrates counteract the oxidative stress and prevent cell damage [20]. Specifically, the water-soluble antioxidants (i.e., ascorbic acid, glutathione, phenols) react with oxidants in the cytosol, while the fat-soluble antioxidants (carotenoids, vitamin E) avoid the peroxidation of cell membranes. In our experiments, we observed a reduction over time in the soluble antioxidant content until 60′ from HL stress, suggesting a progressive depletion and utilization of those compounds in the defense mechanisms. We suppose that the considerable employment of water- and fat-soluble antioxidants was sufficient to avoid the excessive load of ROS in remote tissues preserving them by irreversible damages.

Different mechanisms are involved in the defense against abiotic stresses. The poly(ADPribose)-polymerases (PARPs) are enzymes with a proven key role in the protection against the harmful effects of environmental stress through down- or up-regulation of their activity [12,21]. However, in what way the reduction of PARP activity may enhance the stress tolerance is, to date, still poorly understood. In our study, the direct exposure of inducing top leaves to HL promoted an increase of PARP activity compared to Control, which remained high up to 60′ from the beginning of light stress. Conversely, the decline of soluble antioxidant capacity may be explained considering that an elevated PARP activity requires high consumption of NAD(P), limiting the NAD(P)H availability essential to regenerate the reduced forms of antioxidants. Only after 120′ from light stress, the PARP activity was down-regulated while the soluble antioxidants content increased to the values measured before HL stress. Our results are consistent with previous papers, which demonstrated that the down-regulation of PARP activity enhances plant stress tolerance [22,23] while PARP expression and activity is regulated by cellular redox homeostasis. In our experiment, the “inducing” leaves exposed to HL for a long time likely acclimated to light stress by an improved synthesis of soluble antioxidants and PARP activity reduction. On the contrary, a different acclimation mechanism to light stress may be supposed in the “receiving” leaves (systemic tissues). In the latter, the PARP activity was sustained until “inducing” leaves were exposed to light stress of 60′, and contextually, the antioxidant content surpassed the pre-stress values. When the “receiving” leaves were exposed to light stress directly, both PARP activity and water- and fat-soluble antioxidants decreased. We hypothesize a link between the soluble antioxidants pool and PARP activity in the acclimatized “receiving” leaves. PARP is a redox-sensitive enzyme whose activity could be regulated by soluble metabolites and, in particular, by the reduced glutathione (GSH) in the nucleus via simple thiol–disulphide exchange mechanisms or through glutathionylation, and an increase in GSH could occur to offset cellular oxidation caused by enhanced oxidation of the NAD pool [24]. We supposed that in the acclimatized “receiving” leaves, an increase in the soluble antioxidant synthesis, and, in particular, an enhancement in reduced glutathione, occurred. The “receiving” leaves, when directly exposed to HL, would consume antioxidants to counteract the ROS overproduction and down-regulate the PARP activity to increase the tolerance to light stress.

An excessive NAD depletion by PARP alters the cell homeostasis leading to the apoptosis of cells. It is possible that in “receiving” leaves, the strong consumption of antioxidants to neutralize ROS, and the high NAD^+^ utilization by PARP after 60′ of stress, would determine the synthesis of soluble antioxidants via alternative pathways such as the γ-glutamyl cysteine (γGluCys) synthetase and GSH synthetase, or Smirnoff–Wheeler. In particular, this latter is the dominant pathway for AsA synthesis in photosynthetic tissues [25], while tocopherol is regenerated from AsA [26].

The acclimation process in the systemic tissues (receiving leaves) induced a different regulation of PSII photochemistry, which appeared strongly affected by the duration of stress on the “inducing” leaves. The light-stressed receiving leaves were strongly influencedonly after the inducing leaf was exposed to a PPFD of 2000 μmol m^−2^ s^−1^ for 5′, as indicated by the low Φ_NO_*/*Φ_NPQ_ and F_v_/F_m_ ratios. A low ratio of Φ_NO_ to Φ_NPQ_ may be beneficial in preventing damages and may preserve the photochemical yield under high light stress [27]. Our data indicate that the tolerance to light stress of “receiving” leaves increased with the increasing of the stress duration on “inducing” leaves. Indeed, after “inducing” leaves were exposed to HL for 120′, the systemic tissues (receiving leaves) showed values of electron transport rate and Φ_NO/_Φ_NPQ_ ratio comparable to those found in the “inducing” leaves, indicated that both inducing and receiving leaves exhibited a similar vulnerability to light stress. It is noteworthy that the bottom “receiving” leaves, located down on the stem, get a reduced amount of irradiance during the development compared to the apical top “inducing” leaves, and for this reason, in the absence of any systemic signals, should be more vulnerable le to photoinhibition.

Overall, our data suggest that a response–acclimation pathway is activated in remote tissues of bean plants not yet subjected to stress. This pathway likely involves a ROS wave, which would trigger the PARP enzyme in a mechanism still to be clarified. We hypothesize a link between water- and fat-soluble antioxidants and PARP activity in both local and remote tissues involved in the systemic acquired acclimation (SAA). The increased tolerance in plants exposed to HL implicates an increased synthesis of soluble antioxidants while the PARP activity is reduced to limit excessive consumption of NAD(P).

## 4. Materials and Methods

### 4.1. Plant Growth Conditions

Seeds of *Phaseolus vulgaris* L. were sown in 5 L plots filled with soil (86% peat, 9% sand, 3% quartz sand, 2% perlite) and kept in the dark until germination. After germination, seedlings were moved in a growing chamber under controlled environmental parameters: air temperature 25–15 °C day/night, relative humidity 65/85% day/night, photoperiod 16–8 h day/night. The Photosynthetic Photon Flux Density (PPFD) at the top of canopy was 200 ± 10 μmol photons m^−2^ s^−1^ and 90 ± 10 μmol photons m^−2^ s^−1^ at the bottom of canopy. Plants were irrigated two times per the week and fertilized weekly by a nutrient solution (N:K:P 20:20:20,Gesal Techno-Pro). The total of cultivated potted plants was 20. The plants were divided into four groups for the following experiments.

### 4.2. Experimental Procedure

To pursue our aims, the experimental procedure provided three distinct experiments with four groups of plants to avoid any possible interference due to multiple stresses on the same plant.

#### 4.2.1. First Experiment

**Figure 6:**
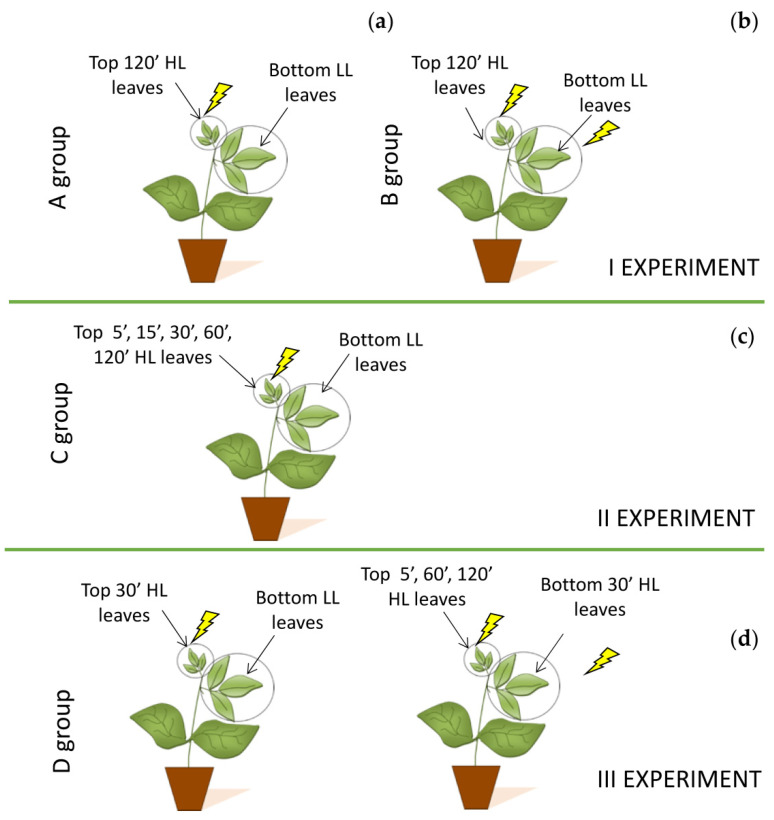
Scheme of the three experiments: First experiment (**a**) In the A plant group, Top HL “inducing” leaves were exposed for 120′ to 2000 PPFD, while the Bottom LL “receiving” leaves were kept for the same duration of time at growth irradiance of 90 PPFD. (**b**) In the B plant group, Top HL “inducing” leaves were exposed for 120′ to 2000 PPFD, while the Bottom HL “receiving” leaves were exposed for 120′ to 2000 PPFD. Second experiment (**c**): In C plant group Top HL leaves were exposed for 5′, 15′, 30′, 60′, and 120′ to 2000 PPFD, while the Bottom LL leaves were kept for the same duration of time at growth irradiance of 90 PPFD. Third experiment (**d**): Finally in the D plant group, Top HL leaves were exposed for 30′ at 2000 PPFD, while the Bottom LL leaves remained unstressed. After Top HL leaves were exposed for 5′, 60′, and 120′ to 2000 PPFD, while the Bottom HL leaves were exposed for 30′ to 2000 PPFD.

For the first experiment, bean plants were divided in two groups: Group A and Group B, each composed of five plants (Figure 6). In plant group A, Top “inducing” leaves (positioned at the top of the stem) were subjected to high light stress and exposed for 120′ to 2000 μmol photons m^−2^ s^−1^ (Top HL leaves). Contextually, on the same plant individual, bottom “receiving” leaves (located at bottom of the stem) were kept at growth light condition of 90 ± 10 μmol photons m^−2^ s^−1^ (Bottom LL leaves). At the end of 120′, both five top and five bottom leaves were collected from five different plants, and the PARP activity and total water- and fat-soluble antioxidant capacities were determined (Figure 6a). In plant group B, top leaves were exposed for 120′ to high light stress of 2000 μmol photons m^−2^ s^−1^ (Top HL leaves) and, successively, also the bottom leaves were exposed to a light stress of 2000 μmol photons m^−2^ s^−1^ for 120′ (Bottom HL leaves). After stress, only the bottom HL leaves (n = 5) were collected from five different plants (one pair fromeach plant) for PARP activity and antioxidant capacity determination (Figure 6b).

#### 4.2.2. Second Experiment

In another plant cluster composed of five plants noted as Group C, Top “inducing” leaves were exposed to a light stress of a different duration, applying for 5′, 15′, 30′, 60′, and 120′ a PPFD of 2000 μmol photons m^−2^ s^−1^ on leaf surface (Top 5′, 15′, 30′, 60′, and 120′ HL leaves). Contextually, bottom LL “receiving” leaves were kept at growth irradiance (90 μmol photons m^−2^ s^−1^) (Bottom LL leaves) (Figure 6c). At the end of each stress period, five top HL and five bottom LL leaves from five different plants (one pair from each plant) were collected for biochemical analyses.

#### 4.2.3. Third Experiment

The third experiment was performed to assess if the light stress applied on Top “inducing” leaves may trigger a signal toward the Bottom “receiving” leaves that increase the tolerance of photosynthetic apparatus to light stress once they were exposed directly to high irradiances.

For this purpose, we selected five other plants (Group D). In the first part of the third experiment, Rapid Light Curves (RLCs) were performed on five unstressed Top leaves, grown at the irradiance of 200 μmol photons m^−2^ s^−1^ and five unstressed Bottom leaves grown at the irradiance of 90 μmol photons m^−2^ s^−1^. After, only top “inducing” leaves were exposed to a light stress of 2000 μmol photons m^−2^s ^−1^ for 30′ (Top HL leaves), and RLCs were repeated, while the Bottom LL “receiving” leaves (Bottom LL leaves) were kept at growth irradiance. In the second part of the experiment, once Top leaves were stressed for 5, 60, and 120 min to 2000 μmol photons m^−2^ s^−1^ (Top 5′, 60′, and 120′ HL leaves), also Bottom LL leaves were stressed for 30′ at 2000 μmol photons m^−2^ s^−1^ (Bottom HL leaves) and analysed by RLCs (Figure 6c).

### 4.3. Chlorophyll Fluorescence Measurements

Rapid light curve (RLC) (0, 125, 190, 285, 420, 625, 820, 1150, 1500 μmol photons m^−2^ s^−1^) were performed by using a portable Pulse Amplitude Fluorometer (Junior-PAM, Walz, Germany) on unstressed control, and on stressed top and bottom leaves after leaf exposure to 2000 μmol photons m^−2^ s^−1^ for 30′. On bottom stressed leaves, RLC were performed after exposing the top leaves to 2000 μmol photons m^−2^ s^−1^ for 5′, 60′ and 120′. The partitioning of absorbed light energy was estimated according to Kramer et al. [21,28]. The quantum yield of PSII electron transport was estimated following Genty et al. [29] as Φ_PSII_ = (F′_m_ − F_s_)/F′_m_. This parameter was utilized to calculate the quantum yield of the regulated energy dissipation (Φ_NPQ_) and of the non-regulated energy dissipation (Φ_NO_) following the formulas: Φ_NPQ_ = 1 − Φ_PSII_ − 1/(NPQ + 1 + qL × (F_m_/F_0_ − 1)) and Φ_NO_ = 1/(NPQ + 1 + qL × (F_m_/F_0_ − 1)), respectively. The coefficient of photochemical quenching, qL, was estimated as reported in Kramer et al. [28]. The electron transport rate (J_f_) was calculated as Φ_PSII_ × PPFD × 0.5 × 0.84 [30], considering the absorbed photon energy equally distributed between PSI and PSII and 0.84 the assumed light absorbance of the leaf.

The maximum photochemical efficiency (F_v_/F_m_) of PSII was determined on 30′ dark-adapted leaves as F_v_/F_m_ = (F_m_ − F_0_)/F_m_. This time is sufficient to allow a complete relaxation of reaction centres [31].

### 4.4. H_2_O_2_ Determination

The H_2_O_2_ levels were measured following the method described by Zhou et al. [32]. Briefly, 0.3 g of leaves were grounded in a mortar with a pestle, in the presence of 3 mL of 5% Trichloroacetic acid (TCA) and 0.10 g activated charcoal. The homogenate was centrifuged at 10,000 rpm for 20 min at 4 °C. The supernatant was adjusted to pH 8.4 with 17 M ammonia solution and then filtered. Then, a reaction mixture containing 1 mL of filtrate, 8 µg of catalase and 1 mL of colorimetric reagent was incubated for 10 min at 30 °C. The absorbance of the samples was determined spectrophotometrically at 505 nm. The colorimetric reagent contained 10 mg of 4-aminoantipyrine, 10 mg of phenol, 5 mg of peroxidase (150 U mg^−1^), dissolved in 50 mL of 100 mM acetic acid buffer (pH 5.6). The content of H_2_O_2_ was calculated using a standard curve and expressed as µmol H_2_O_2_ g^−1^ fresh weight of tissue.

### 4.5. Nuclei Isolation

Nuclei isolation was performed according to Arena et al. [22]. All operations were carried out on ice or at 4 °C. All dwarf bean leaves (1 g) were harvested, cut and resuspended in 10 mM Tris-HCl pH 8.0, 1 mM EDTA, 1 mM EGTA, 1 mM PhMeSO_2_F, 10 mM MgCl_2_, 5 mM β-mercaptoethanol, 0.5% NP 40 and 2 μgmL^−1^proteases inhibitors cocktails (1:4, *w*/*v*)/buffer A). The samples were homogenized for 15–30 s at low speed by an Ultra Turrax T8 (IKA-WERKE). The homogenates were filtered through three layers of cheesecloth, and the filtrate was centrifuged at 1500× *g* for 30 min at 4 °C (Rotor Beckman JA-25.50). The pellets containing nuclei were suspended in buffer A and centrifuged three times, as described above. Finally, the pellets (nuclear fractions) were suspended in a small volume of buffer A containing 2% glycerol, without NP 40 and β-mercaptoethanol. Protein content was determined by Bradford’s reagent (BioRad, Chicago, IL, USA) according to the provided instructions.

### 4.6. SDS-PAGE and Western Blotting

Electrophoretic analyses of all the leaves’ nuclear fractions (20 µg) were performed on 10% polyacrylamide slab gels in the 0.025 M Tris-0.192 M glycine-0.1%SDS buffer, pH 8.3 at 18 mA [15]. The gel was stained in silver, according to the procedure described by Rabilloud [33]. Gel was fixed in 30% methanol and 10% acetic acid under stirring overnight. It was then treated with a sensitization solution containing 0.5 M potassium acetate, 25% methanol, 0.1 mM potassium tetrathionate, and 0.5% glutaraldehyde, stirred for 1 h then washed in water for at least 40 min before being placed in 0.2% silver nitrate and 0.05% formaldehyde in the dark for 30 min. After being rinsed in water, it was developed in 3% sodium carbonate, 1% sodium thiosulfate, and 0.025% formaldehyde. The reaction was stopped with 4.12 mM Tris-100% acetic acid buffer.

For immunoblotting, electrophoresed proteins were transferred onto polyvinylidene fluoride (PVDF) filter (0.45 µm; Cat No. IPVH00010, Merck Millipore, Milano, Italy) by a Bio-Rad Transblot system at constant 200 mA in 0.025 M Tris-0.192 M glycine buffer, pH 8.6, containing SDS 0.025% at 4 °C for 2 h. The PVDF filter underwent repeated washes with 50 mM Tris-HCI buffer, pH 8.0, and 150 mM NaCl (TBS), containing 0.5% Tween 20, and then treated in TBS-0.5% Tween, containing 3% gelatine at room temperature for 2 h, to saturate the non-specific bond sites.

Thereafter, the filter was incubated with polyclonal primary anti-PARP antibodies (H-250, Santa Cruz, CA, USA 1:1000) in 0.05% TBS-Tween and 0.3% gelatine at room temperature for 2 h. After several washes in TBS-0.05% Tween, at room temperature for 1 h, it was incubated with peroxidase (HRP)-conjugated goat anti-rabbit (Cat No. 31460, Life Technologies, Monza, Italy) directed against the primary antibodies.

Finally, the filter underwent a series of washes before detecting peroxidase activity using a kit for chemiluminescence (ECL Western Blotting Substrate, Pierce, 32106, Waltham, MA, USA). The acquisition and analysis of the images were carried out by Chemidoc (Bio-Rad) and the Quantity One program.

### 4.7. PARP Activity

The enzyme activity was assayed as described by Vitale et al. [23]. The reaction mixture (final volume 50 μL) contained 0.5 M Tris-HCl, pH 8.0, 50 mM MgCl_2_, 10 mM DTT, 0.4 mM [^32^P]NAD^+^ (10,000 cpm/nmole) and a defined amount of proteins (20 µg). After incubation for 15 min at 25 °C, the reaction was stopped by adding ice-cold 30% trichloroacetic acid (*w*/*v*). The mixture was filtered on Millipore filters (HAWPP0001, 0.45 μm pore size, MF-Millipore, Milan, Italy) and washed with 7% trichloroacetic acid. The activity was measured as acid-insoluble radioactivity by liquid scintillation in a liquid phase scintillator (Beckman LS 1701, Beckman Coulter, Milan, Italy). The enzyme activity is expressed in enzymatic milliunit, defined as the amount of enzyme required to convert 1 nmol of NAD+ per minute under standard conditions.

### 4.8. Determination of Total-Soluble and Fat-Soluble Antioxidant Capacity

The total-soluble antioxidants were extracted in a mixture consisting of methanol, water, and formic acid (80:20:0.1) for 3 h in the dark. The hydrophilic extract (S1), represented by the supernatant obtained after centrifugation at 3500 rpm for 1 min, was stored at 4 °C. The pellet was subjected to two subsequent extractions. The hydrophilic extracts S1, S2, and S3, were combined and further centrifuged at 10,000 rpm for 5 min at 4 °C. The fat-soluble antioxidants were extracted in acetone by performing the same experimental procedure described above.

Free radical scavenging activity of the total-soluble and fat-soluble extracts was determined by 2,2′-azino-bis(3-ethylbenzthiazoline-6-sulphonic acid) (ABTS•+) radical cation decolorization assay, as described by Re et al. [34], with slight modifications. ABTS•+ cation radical was obtained by the reaction between 7 mM ABTS and 2.45 mM potassium persulfate (1:1). The reaction mixture was stored for 16 h in the dark at room temperature before use and utilized within two days. The ABTS•+ solution was diluted with methanol to obtain an absorbance of 0.700 ± 0.050 at 734 nm. Then 100 μL of plant extract was added to 3.900 mL of diluted ABTS•+ solution, and the absorbance was measured at 734 nm. An appropriate solvent blank was run in each assay. All the measurements were carried out at least three times. The results were compared to a calibration curve using Trolox as standard (0–15 μM) and were expressed as μmol Trolox g^−1^ fresh weight of tissue.

### 4.9. Statistical Analysis

The statistical analysis of the data was performed by one-way ANOVA. The package Sigma-Plot 12.0 (Jandel Scientific, San Rafael, CA, USA) was used for statistical data processing. Shapiro–Wilk and Kolmogorov–Smirnov tests were used to check for normality. The Holm–Sidak post-hoc test was applied for all multiple comparison procedures based on a significance level of *p* ≤ 0.05.

## Figures and Tables

**Table 1:** Maximum photochemical efficiency (F_v_/F_m_) in Top inducing leaves (Control and 30′ HL stressed) and bottom receiving leaves (Control and 30′ HL stressed). The bottom receiving leaves were subjected to 30′ HL stress and measured after top inducing leaves were exposed to 5′, 60′, and 120′ HL stress.

Top Inducing Leaf		Bottom Receiving Leaf			
Control	30′ HL stressed	Control	30′ HL stressed (after 5′ HL stress of top leaves)	30′ HL stressed (after 60′ HL of top leaves)	30′ HL stressed (after 120′ HL stress of top leaves)
0.809 ± 0.005 ^a^	0.746 ± 0.020 ^b^	0.808 ± 0.004 ^a^	0.615 ± 0.036 ^c^	0.734 ± 0.012 ^b^	0.742 ± 0.017 ^b^

Different letters indicate statistically significant differences among treatments (*p* ≤ 0.05) by One-Way ANOVA followed by Holm–Sidak post-hoc test.

## Data Availability

The data presented in this study are available on request from the corresponding author.

## Supplementary Materials

The following supporting information can be downloaded at: https://www.mdpi.com/article/10.3390/plants11141870/s1, **Figure S1**: 10% SDS-PAGE (a) and immunoblotting with anti-PARPH250 antibodies (b). Control (nuclear fraction of leaves Control); Top HL (nuclear fraction of “inducing” leaves stressed for 120′); Bottom LL (nuclear fraction of unstressed “receiving” leaves); Bottom HL (nuclear fraction of “receiving” leaves stressed for 120′); **Figure S2**: 10% SDS-PAGE (a) and immunoblotting with anti-PARP H250 antibodies (b): Control (nuclear fraction of Control leaves); Top HL 5′ (nuclear fraction of “inducing” leaves stressed for 5 min); Bottom LL 5′ (nuclear fraction of unstressed “receiving” leaves for 5′); Top HL 15′(nuclear fraction of “inducing” leaves stressed for 15 min); Bottom LL 15′(nuclear fraction of unstressed “receiving” leaves for 15′); Top HL 30′ (nuclear fraction of “inducing” leaves stressed for 30 min); Bottom LL 30′ (nuclear fraction of unstressed “receiving” leaves for 30′); Top HL 60′ (nuclear fraction of “inducing” leaves stressed for 60′); Bottom LL 60′(nuclear fraction of unstressed “receiving” leaves for 60′).
