# Supplementary material for: Role of Poly(ADP-Ribose) Polymerase (PARP) Enzyme in the Systemic Acquired Acclimation Induced by Light Stress in Phaseolus vulgaris L. Plants
Source: Plants (Basel). 2022 Jul 18;11(14):1870. doi: 10.3390/plants11141870 (PMC9316121; doi:10.3390/plants11141870)
Supplement: Supplementary file 1 [file plants-11-01870-s001.zip › plants-1788015-supplementary.pdf]

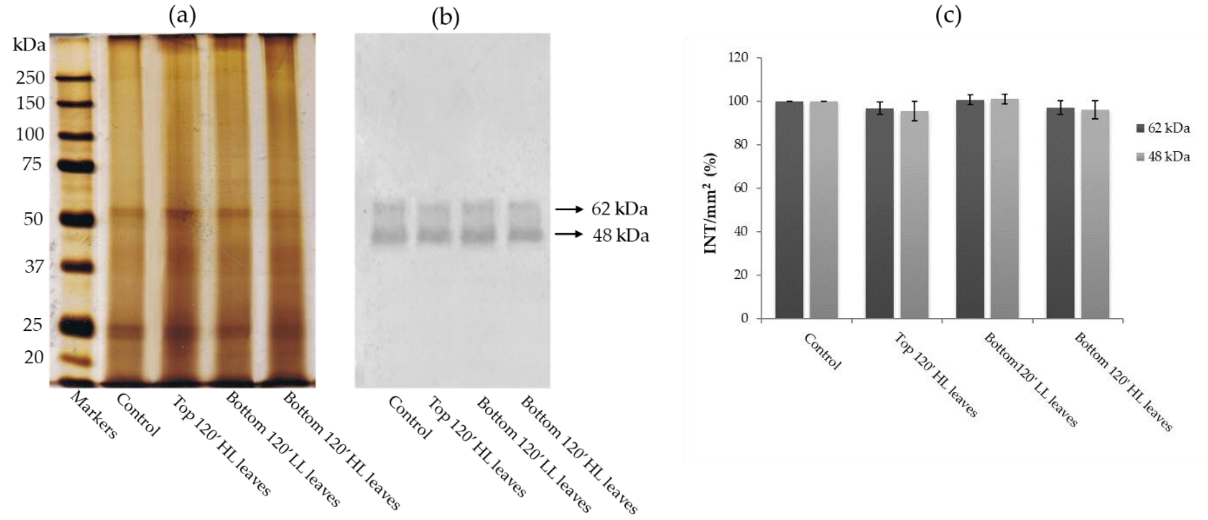

Figure S1. 10% SDS-PAGE (**a**), immunoblotting with anti-PARPH250 antibodies (**b**) and percentual densitometric analysis of 62 kDa and 48 kDa signals (**c**): Control (nuclear fraction of leaves Control); Top 120′ HL leaves (nuclear fraction of "inducing" leaves stressed for 120′); Bottom LL (nuclear fraction of unstressed "receiving" leaves for 120′); Bottom 120′ HL leaves (nuclear fraction of "receiving" leaves stressed for 120′). The bars represent the mean (± SE) of the INT/mm^2 (%) of each sample compared to the control, considered 100%. No statistically significant percentage difference was observed among all examined nuclear fractions ($p \leq 0.05$) using the Mann-Whitney U test.

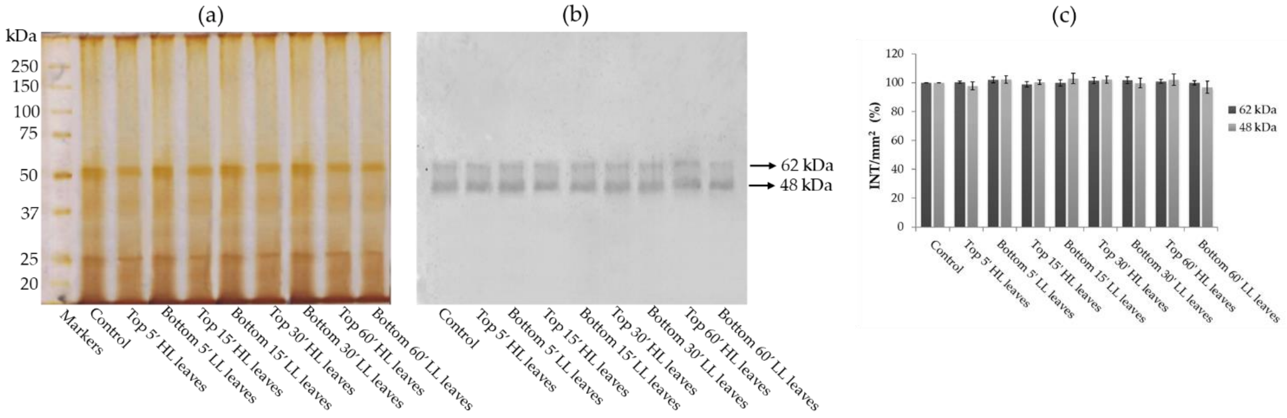

Figure S2. 10% SDS-PAGE (**a**) immunoblotting with anti-PARP H250 antibodies (**b**) and percentual densitometric analysis of 62 kDa and 48 kDa signals (**c**) of Control, Top and Bottom leaves at different timing of stress: Control - nuclear fractions of unstressed *Phaseolus vulgaris* L. leaves; Top 5′ HL leaves - nuclear fraction of “inducing” leaves after 5′ stress; Bottom 5′ LL leaves (nuclear fraction of unstressed “receiving” leaves); Top 15′ HL leaves - nuclear fraction of “inducing” leaves after 15′ stress; Bottom 15′ LL leaves - nuclear fraction of unstressed “receiving” leaves; Top 30′ HL leaves (nuclear fraction of “inducing” leaves after 30′ stress); Bottom 30′ LL leaves - nuclear fraction of unstressed “receiving” leaves; Top 60′ HL leaves - nuclear fraction of “inducing” leaves after 1h stress; Bottom 60 ‘LL leaves - nuclear fraction of unstressed “receiving” leaves).The bars represent the mean (± SE) of the INT/mm^2 (%) of each sample compared to the control,

considered 100%. No statistically significant percentage difference was observed among all examined nuclear fractions ($p \leq 0.05$) using the Mann-Whitney U test.
